# A Novel Approach, Based on the Combined Action of Chitosan Hydrogel and Laccases, for the Removal of Dyes from Textile Industry Wastewaters

**DOI:** 10.3390/gels9010041

**Published:** 2023-01-04

**Authors:** Filomena Sannino, Elena Di Matteo, Mariarosaria Ambrosecchio, Domenico Pirozzi

**Affiliations:** 1 Department of Agricultural Sciences, University of Naples Federico II, Via Università 100, Portici, 80055 Naples, Italy; 2 Laboratory of Biochemical Engineering, Department of Chemical Engineering, Materials and Industrial Production (DICMaPI), University of Naples Federico II, Piazzale Tecchio 80, 80125 Naples, Italy

**Keywords:** biopolymer, crosslinking, adsorption/degradation of dyes, laccase onto chitosan hydrogel, reusability

## Abstract

Dyes are considered as one the most important classes of contaminants that threaten the environment and human life. The synergy between the adsorption capacity of chitosan hydrogels and the catalytic properties of the enzyme laccase was exploited to improve the removal of contaminants from a liquid stream. The adsorption capacity of a chitosan hydrogel was tested on three different textile dyes. The effect of pH on the adsorption efficiency was dependent on the dye tested: the removal of methylene blue (MB), a cationic dye, was more effective at alkaline values of pH, whereas bromophenol blue (BPB) and Coomassie brilliant blue (BB), both anionic dyes, were more effectively removed under acid environments. The use of laccase immobilized onto chitosan has significantly improved the efficiency of dye removal, exploiting the synergy between the adsorption capacity of chitosan and the catalytic properties of the enzyme. The simultaneous processes of adsorption and enzymatic degradation improved the dye removal whatever the pH value adopted, making the removal efficiency less dependent from the pH changes. The chitosan used as a support for the immobilization of laccases showed good stability under repeated cycles, demonstrating the feasibility of the method developed for the application in wastewater remediation.

## 1. Introduction

The deterioration in the quality of water resources is an increasingly serious problem worldwide [1] for two main reasons: (i) water resources are being progressively reduced due to the growth of the population, climate change, and the consumption of water deriving from agricultural and industrial activities [2]; (ii) the inappropriate discharge of different pollutants such as pesticides and drugs, as well as the lack of information about their treatment methods [3].

Consequently, aquatic environments are undergoing a progressive degradation, causing a drastic reduction in the quantity of available water that is safe to use, and worsening the water crisis [4]. A typical example of these dangerous pollutants is dyes, released into the environment through different printing and textile activities [5,6]. They are complex organic molecules that are difficult to break down. Due to their intense coloring, the presence of these dyes in water bodies prevents the penetration of light, which results in a reduction in photosynthetic activity, inhibiting the growth of aquatic biota [7].

The conventional methods commonly used in the treatment of effluents containing dyes, such as membrane filtration, coagulation/flocculation, chemical precipitation, and biological degradation, are often energy-consuming, and are affected by slower response and reduced sensitivity [8]. Furthermore, the efficiency of these methods is reduced in the presence of unstable colloidal matter [9], as well as when the load of organic matter is high. In addition, some of these methods lead to the generation of toxic by-products [10]. 

Adsorption offers a promising approach to develop effective methods for the removal of dyes from wastewaters [11,12,13,14,15], being less expensive and requiring simple operation systems. In addition, it can be carried out by using a wide range of adsorbent materials, both synthetic or natural, allowing for the physical or chemical adsorption efficiency with both organic and inorganic pollutants. Consequently, both academic and the industrial research are focused on the development of new adsorbent materials of applicative interest. 

Chitosan, a polysaccharide made of randomly distributed D-glucosamine and N-acetyl-D-glucosamine, is attracting growing interest as a sorbent material, for the following reasons: (i) it offers a high adsorption efficiency, as it possesses hydroxyl groups (–OH) and primary amine groups (–NH_2_), both acting as adsorption sites and also allowing for high potential for diverse types of modifications [16]; (ii) the presence of these functional groups enables the interaction of chitosan with different compounds to obtain composite materials having improved adsorption properties; (iii) the use of chitosan is consistent with the principles of the circular economy, as it is obtained from alkaline or enzymatic N-deacetylation of chitin, a residual product of shrimp and seafood processing [17]. The annual production of chitin is about 1 × 10^12^ tonnes. Being insoluble in aqueous solution, chitin hardly interacts with other compounds, and this limits its industrial application [18]. Consequently, the conversion of chitin into chitosan prevents the disposal of chitin as a waste.

In the recent years, chitosan has been used for applications in different fields (food, medicine, cosmetics, and wastewater treatment) due to its unique and attractive characteristics, including biocompatibility, low cost, nontoxicity, mechanical stability, antimicrobial activity, and biodegradability [19]. In order to extend its applicability, chitosan has been modified with a variety of other additives [20] to form composites or hybrids to improve its surface area, to extend the range of pollutants potentially adsorbed, and to allow for an easier separation from the aqueous phase (e.g., producing magnetic chitosan by interaction with Fe_3_O_4_). 

In particular, chitosan hydrogels are raising a growing interest for their use as adsorbents. Hydrogels can be produced under mild conditions, using cheaper natural polymers such as chitosan, starch, cellulose, alginate, and pectin [21,22]. In particular, chitosan hydrogels offer improved mechanical, thermal, and porosity properties, as well as higher adsorption capacity in comparison with native chitosan [23,24].

So far, the properties of hydrogels as adsorbents have been improved by combining them with different types of additives, such as metals, nonmetals, metal oxides, and polymeric moieties [21]. Additives can be used for different purposes; for example, graphene has been employed to exploit its unique chemical, thermal, and mechanical properties [25], whereas biochar increases both the specific surface and the mechanical resistance [26], L-glutathione has been exploited to change the surface charge of the adsorbents by addition of new functional group [27].

The properties of hydrogels can also be improved by using them as matrices for enzyme immobilization. Hydrogels are ideal candidates as supports for immobilized enzymes, in that their large surface area, their elasticity, and their tunable functional properties contribute to increase the loading of enzyme, as well as its catalytic activity [28,29].

So far, chitosan-based hydrogels have been used to immobilize different enzymes [28]. In some instances, chitosan-based hydrogels have also been used as solid supports for laccases [30,31,32]. However, in these works, laccase has been used to increase the antibacterial activity of the chitosan [31,32].

The combined used of laccases and chitosan hydrogels is a relatively unexplored approach to improve the potential of chitosan for wastewater remediation. As a matter of fact, laccase is a versatile oxidase enzyme, able to catalyze the degradation of different pollutants, in particular dyes [33].

It is worth noting that chitosan particles have been shown to be excellent solid supports to improve the catalytic activity and stability of enzymes. Several investigations reported the successful immobilization of laccase on the surface of nanomaterials, to improve their stability and catalytic properties for the removal of dyes from waters. For instance, free laccase was immobilized with improved properties and reused on the surface of ZnO and MnO nanoparticles for the degradation of alizarin dye [34]. Magnetic nanoparticles are the most used and favored as support for enzymes, due to their magnetic behavior that allows for separation from the reaction medium, using an external magnet. Unlike other oxidases, such as peroxidases, the laccase-catalyzed degradation of industrial dyes does not require the addition of H_2_O_2_, offering an important applicative advantage. In this paper, the synergy between the adsorption capacity of chitosan hydrogels and the catalytic properties of the enzyme laccase is exploited to improve the removal of contaminants from a liquid stream. The laccases (polyphenoloxidase, EC 1.10.3.2) are multicopper oxidase enzymes that have raised growing interest in recent years, as they are cheaper and significantly stable under process conditions. In addition, their low substrate specificity allows for the degradation of a wide range of contaminants, such as phenols, aromatic amines, and some inorganic compounds. Three different dyes—methylene blue (MB), bromophenol blue (BPB), and Coomassie brilliant blue (BB)—widely employed in the textile industry, have been used as model compounds to test the efficiency of a novel approach based on the simultaneous adsorption and enzymatic catalysis. The selected compounds are representative of two different classes of dyes: MB is a heterocyclic dye, whereas BPB and BB are triphenylmethanes.

## 2. Results and Discussion

### 2.1. Adsorption of Dyes on Chitosan

**Figure 1:**
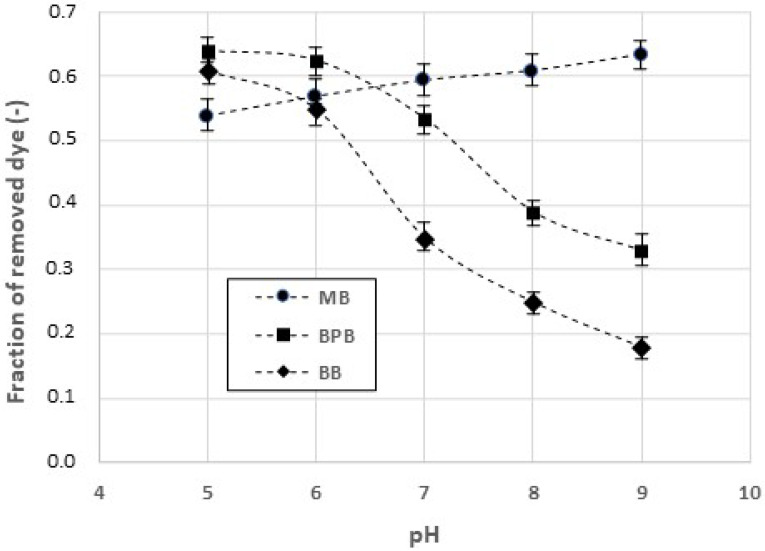
Amounts of methylene blue (MB), bromophenol blue (BPB), and Coomassie brilliant blue (BB) adsorbed on chitosan hydrogel. S/L ratio = 1/100, contact time = 120 min.

A first series of experimental tests was devoted to studying the adsorption of MB, BPB, and BB on chitosan hydrogel particles. Figure 1 shows the amounts of dye adsorbed on chitosan at solid/liquid ratio (S/L) = 1/100 g/g, after a contact time of 120 min, as a function of pH.

The adsorption of MB on chitosan hydrogel shows a weak, progressive increase as the pH increases. On the contrary, the amount of adsorbed BPB is a decreasing function of the pH. Similarly, the amount of adsorbed BB decreases as the pH increases, though the adsorbed amounts are lower in comparison to those observed with BPB. To explain these results, it should be taken into account that the surface charge distribution of both the dyes and the chitosan can be significantly affected by the pH. The chitosan has a point of zero-charge at a pH value of about 7.35 [35], due to the effect of the amine, carboxylic, and hydroxyl functional groups. Consequently, at pH > 7.35, the surface of chitosan acquires an increasingly negative charge, whereas when pH < 7.35, the surface of the chitosan is positively charged. MB is a cationic dye that in aqueous solution can be present in a cationic and neutral form [36] (p. 16). The corresponding speciation diagram, reported in Figure S2 [37] (p. 36) indicates that at pH > 5, the MB is almost completely present in the cationic form. Consequently, as the pH increases, the adsorption of positively charged MB on the negatively charged chitosan will be higher. Correspondingly, as the pH decreases, the adsorption will be reduced due to the electrostatic repulsion forces. In addition, at lower values of pH, the concentration of H+ ions will be higher, and these ions will compete with MB cations for the adsorption sites that are still empty. As far as concerns to the BPB, this dye undergoes a double deprotonization [38] (p. 334), described by two values of pKa (pH 3.0 and pH 4.6, respectively). It can easily be understood that, as the pH increases, an electrostatic repulsion will occur between chitosan and BPB, both of which will be negatively charged. On the contrary, at lower pH, the amine groups of chitosan will be protonated, thus facilitating the electrostatic attractions with the negatively charged BPB. This explains the decreasing curve of the adsorbed fraction as a function of the pH. The behavior of BB is similar to that of BPB. As the pH of the liquid medium changes, the BB undergoes four sequential acid–base transformations [39] (p. 1909) that are described by four values of pKa (pH −0.5, pH 3, pH 7.6, and pH 9.0, respectively). The corresponding speciation diagram [39] (p. 1910) suggests that when the pH value is higher than 2, BB acquires a negative charge. Consequently, the electrostatic repulsion will occur between chitosan and BB, leading to a decrease in the adsorbed amounts at higher values of the pH.

**Figure 2:**
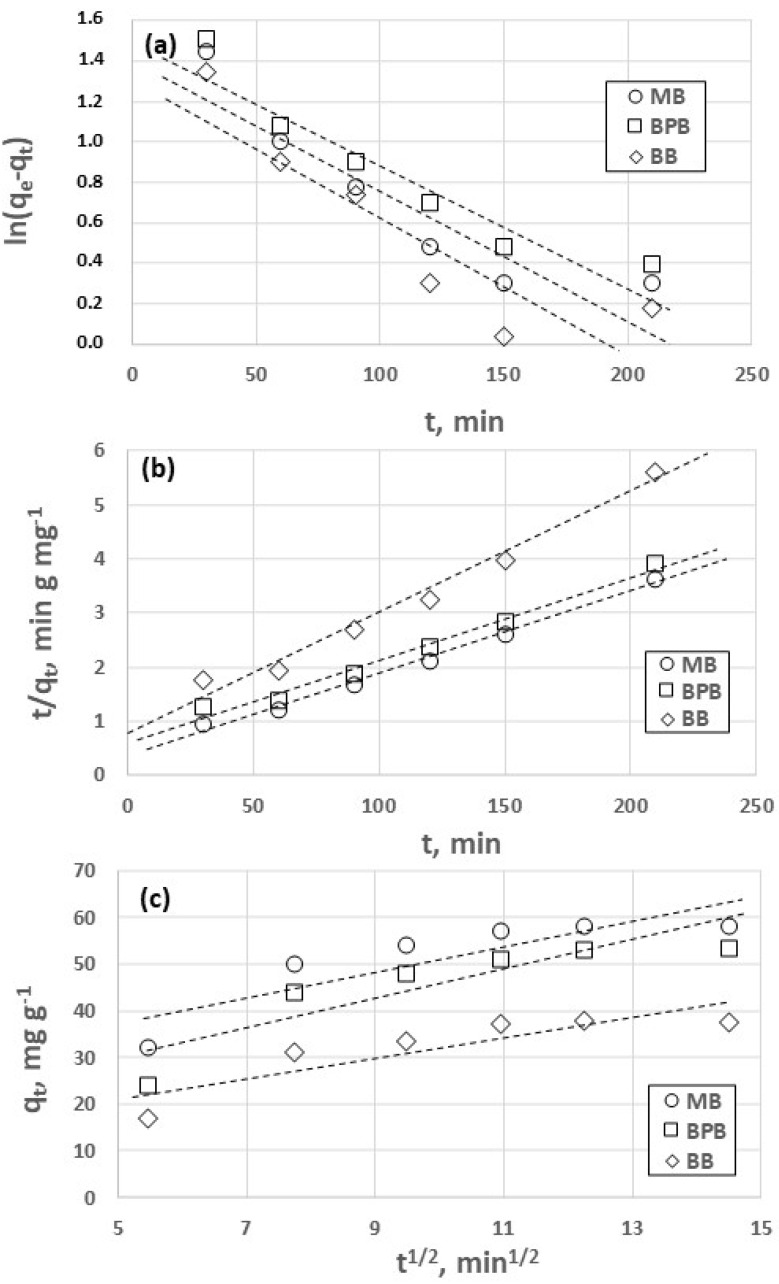
Kinetic models for adsorption of methylene blue (MB), bromophenol blue (BPB), Coomassie brilliant blue (BB) on chitosan hydrogel. S/L ratio = 1/100, contact time = 120 min. Models: PFO (**a**), PSO (**b**), intraparticle diffusion (**c**).

The adsorption kinetics was analyzed, adopting a pH value of 7.0. Three kinetic models were taken into account, namely pseudo-first-order (PFO) pseudo-second-order (PSO), and intraparticle diffusion. The three models are described in paragraph S1. The corresponding fitting plots are reported in Figure 2, whereas the fitting parameters are reported in Table S1. We found the PSO model to be the most suitable to describe the experimental curves, in that the corresponding values of the correlation coefficient (R^2^) were the lowest. This conclusion is confirmed by the observation that the data shown in Figure 2b are better fitted by a linear model, in comparison to those reported in Figure 2a,c. These results are in agreement with previous studies concerning the adsorption of dyes on chitosan-based hydrogels [22,40,41]. 

### 2.2. Degradation of Dyes in the Presence of Laccase 

**Figure 3:**
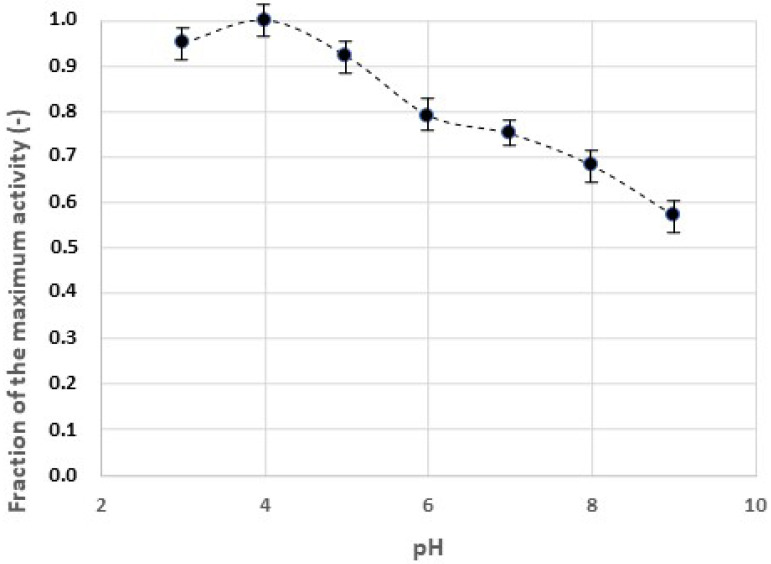
Effect of pH on the catalytic activity of the laccase. Substrate: 5 mM ABTS. T = 37 °C. Reaction time: 10 min.

The effect of pH on the catalytic activity of the laccase in homogeneous solution, measured using ABTS as substrate, is shown in Figure 3. The experimental curve was bell-shaped, with a maximum activity at a pH value of about 4.0, in agreement with the results reported in the literature.

**Figure 4:**
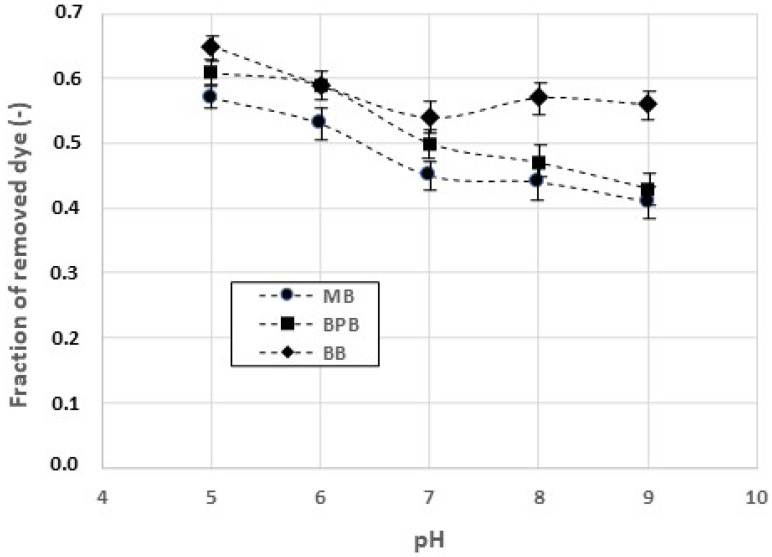
Amounts of methylene blue (MB), bromophenol blue (BPB), and Coomassie brilliant blue (BB) removed by laccases. Reaction time: 120 min.

The soluble enzyme was then tested in the dye degradation. The results obtained with laccases in the presence of MB, BPB, and BB are reported in Figure 4.

The experimental data confirm that the optimal pH of laccase activity is in the acidic range. However, the influence of the pH on the dyes’ removal is more limited in comparison to the effect observed when the dyes are removed by adsorption on chitosan. For all the values of pH adopted, the rate of the enzymatic degradation of BB is slightly faster than those observed when using BPB and MB.

### 2.3. Adsorption of Dyes in the Presence of Laccase Immobilized on the Chitosan Hydrogel

**Figure 5:**
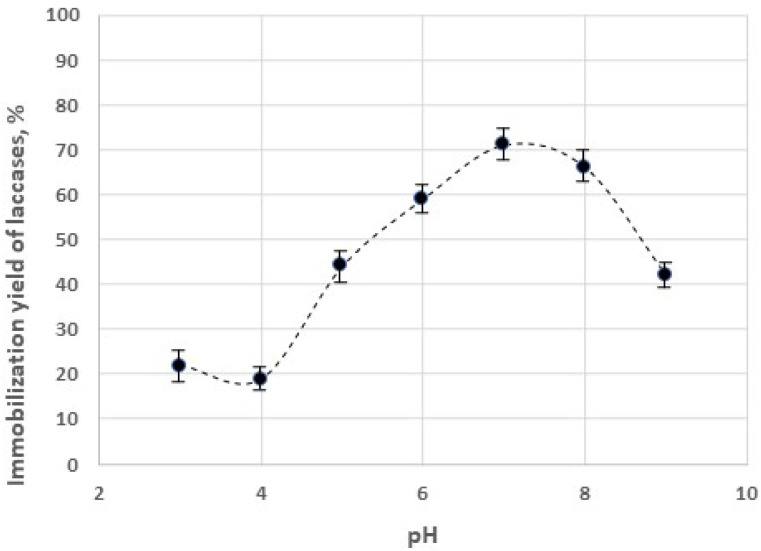
Effect of pH on the immobilization yield of the laccase on chitosan hydrogel. Substrate: 5 mM ABTS.

Laccases were immobilized on chitosan to exploit the synergy between the adsorption capacity of chitosan and the catalytic properties of the enzyme in the remediation of wastewaters. The experimental data in Figure 5 indicate that the yield of immobilization was strongly affected by the value of pH, and that the maximum amount of the immobilized enzyme was obtained under neutral pH values (about 7). Subsequently, the chitosan particles with immobilized laccases were tested in the removal of dyes. The effect of the immobilized enzyme on the efficiency of dye removal can be affected by different factors: it must be taken into account that the enzyme activity is higher at more acidic values of the pH (see Figure 3 and Figure 4), though the maximum immobilization yield of the enzyme is obtained at pH values close to the neutrality. 

**Figure 6:**
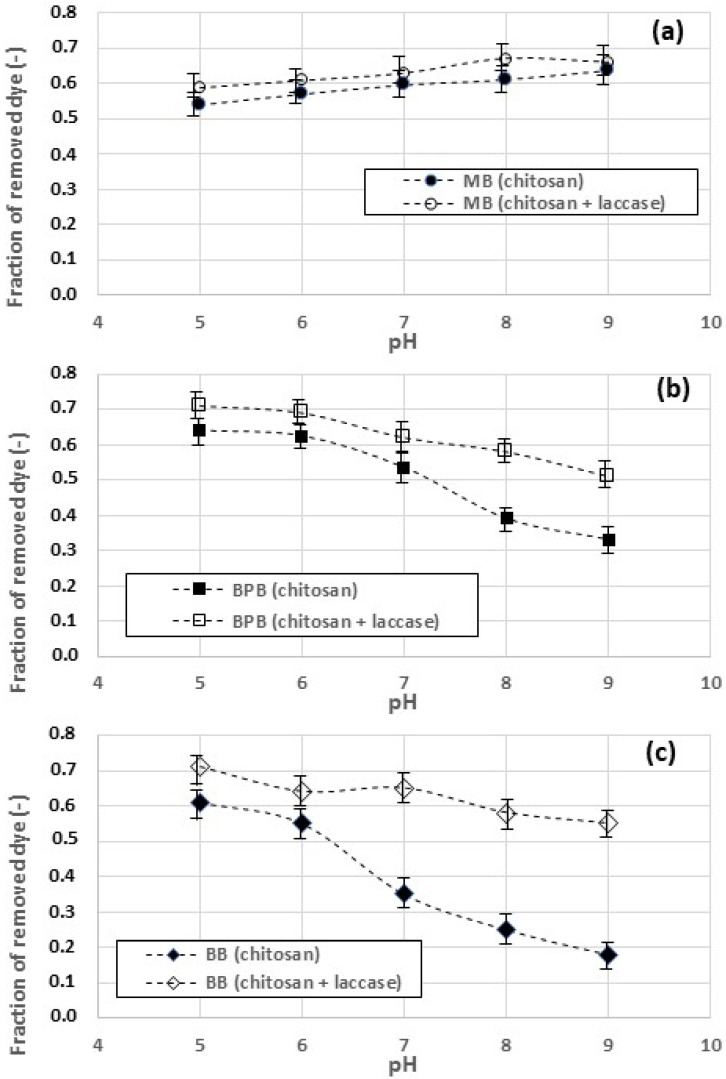
Amounts of dyes removed in the presence of chitosan hydrogel with immobilized laccase: methylene blue (**a**), bromophenol blue (**b**), Coomassie brilliant blue (**c**). S/L ratio = 1/100, contact time = 120 min.

Figure 6a–c compare the dye removal observed using chitosan hydrogel and without laccase. The removal of MB (Figure 6a) is increased in the presence of laccases whatever the pH value adopted, though the effect of the enzyme does not seem pH-dependent. The removal of BPB (Figure 6b) is also improved in the presence of the enzymes, with a more significant increase in the range of pH between 7.0 and 9.0. A similar behavior is observed for the removal of BB (Figure 6c), though in this case, the improvement of dye removal produced by the laccases is more significant. Overall, it can be said that when the chitosan contains immobilized laccases, two main effects are observed: (i) the immobilized laccases improve the dye removal, whatever the pH value adopted; (ii) the overall amount of removed dyes is substantially more uniform as the pH changes.

**Figure 7:**
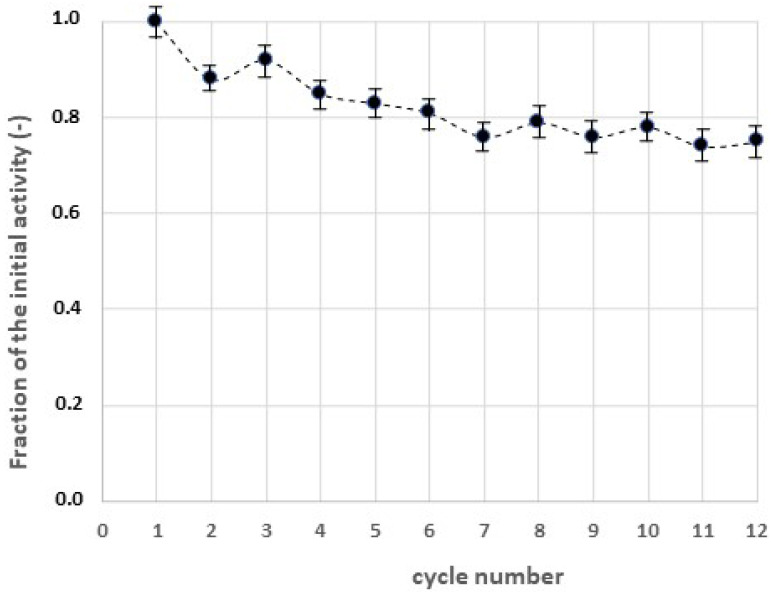
Multiple cycles of adsorption of methylene blue in the presence of chitosan hydrogel with immobilized laccase. S/L ratio = 1/100, contact time = 120 min.

Obviously, important advantages of the enzyme immobilization stem from the long-term stabilization and the possibility of multiple reuse of the catalyst. These are important aspects, as the repeated use of the enzyme is a bottleneck still limiting the large-scale application of the laccase. In this view, we carried out multiple cycles of MB degradation using chitosan hydrogel with immobilized laccase, as shown in Figure 7.

First of all, we observed that the hydrogel nanoparticles retained their mechanical integrity during the multiple-cycle test. This is also due to the use of glutaraldehyde as a crosslinking agent, which generates an improvement in the mechanical properties of the hydrogel [42], yielding a maximum compression of 25.9 ± 1 kPa. Chitosan hydrogel beads with immobilized laccases were also observed using SEM, and no appreciable differences were observed between the morphology of the material before and after each adsorption test.

We also observed a progressive decrease in the removed fraction of MB, probably due to the leakage of the immobilized enzyme, to its long-term inactivation, or to the progressive saturation of the adsorption sites on the surface of the chitosan particles. However, about 75% of the initial removal rate was kept after 12 cycles, demonstrating the feasibility of the method developed in the remediation of the textile wastewaters.

## 3. Conclusions

The experimental data shown demonstrate that chitosan hydrogels are quite versatile and efficient. They can be used for the removal of different textile dyes, though the removal efficiency is significantly affected by the pH of the liquid medium and by the nature of the dyes tested. The immobilization of laccases improved the dye removal whatever the pH value adopted, making the removal efficiency less dependent from the pH changes. The adsorbent with immobilized enzyme showed a good stability after 12 repeated cycles. The stability and the versatility of the chitosan with immobilized laccases demonstrated the feasibility of the method developed for the application in textile wastewater remediation.

## 4. Materials and Methods

### 4.1. Materials 

Low-molecular-weight chitosan (average molecular weight: 110,000, average deacetylation degree: 75%), laccase from Aspergillus spp., methylene blue (MB), bromophenol blue (BPB), Coomassie brilliant blue (BB), ABTS [2,2′-azino-bis-(3-ethyl-benzthiazoline-6-sulfonic acid)], hydroxybenzotriazole (HBT), and all the remaining reactants were purchased from Sigma-Aldrich. All reactants used were analytical-grade.

### 4.2. Synthesis of the Chitosan Hydrogel, Measurement of the Swelling Degree, and FTIR Analysis

Chitosan (2% *w*/*v*) was added to a 1% acetic acid solution, and completely dissolved by 3 h stirring. The solution obtained was added dropwise, using a 1 mm diameter syringe, into a solution of methanol in HCl (20:1 *v*/*v*) containing 1% glutaraldehyde. The hydrogel beads obtained were removed by filtration and used for adsorption experiments.

The obtention of a chitosan hydrogel was testified by measurement of the swelling degree. In this view, the hydrogel beads were allowed to swell in an aqueous solution of acetic acid (pH 5) at 37 °C. Samples of beads were taken at given times and weighted until constant weight. The swelling degree (SD) was measured using the following equation:

SD = W_t_/W_o_

where W_0_ and W_t_ are the initial and final weights of the swollen samples, respectively. 

The swelling degree of the chitosan hydrogel beads used in this study was 56 ± 2%, in agreement with the values reported in previous studies.

The effective formation of the chitosan hydrogel was demonstrated by the FTIR analysis of both the chitosan and the chitosan hydrogel, described in Section 2. As a matter of fact, a detailed comparison of the FTIR spectra demonstrated the formation of a imine groups (C=N) formed by covalent bonding between the free amino groups of chitosan and the aldehyde groups of glutaraldehyde.

### 4.3. Adsorption of Dyes by Chitosan Hydrogel

The adsorption of dyes was carried out at solid/liquid (S/L) ratio of 1/100. In each experiment, 5 mL of dye solution, usually at the concentration of 10 mg/L, was inserted into a 10 mL tube and inserted into an orbital stirrer at 37 °C. Subsequently, 50 mg of chitosan hydrogel particles was added to the solution and kept under stirring (100 rpm). 

At different time intervals, samples were uptaken. The chitosan particles were separated by filtration under vacuum, and the optical density of the solution was measured by a spectrophotometer (Shimadzu UV 1700).

The concentration of each dye before and after the adsorption tests was obtained by absorbance measurement at fixed wavelength values, namely 668 nm for MB, 590 nm for BPB, and 583 nm for BB. The calibration curves obtained for each dye are reported in the following (A is the absorbance, x is the concentration in mg/L):

MB: A = 0.0980 x + 0.0121 R^2^ = 0.982  (λ = 668 nm)

BPB: A = 0.0756 x + 0.0189 R^2^ = 0.991  (λ = 590 nm)

BB: A = 0.0678 x + 0.0101  R^2^ = 0.980  (λ = 583 nm)

In each experiment, the fraction of removed dye was obtained using the following equation:

RF = (C_0_ − C_t_)/C_0_·100 (1)

where C_0_ and C_t_ are the concentrations of dye before and after the adsorption, respectively.

### 4.4. Determination of Laccase Activity

The enzymatic activities of free and immobilized laccase were determined by monitoring the oxidation of 2,2′-azino-bis-(3-ethyl-benzthiazoline-6-sulfonic acid) (ABTS) to its cation radical (ABTS^+^) at 420 nm. The molar extinction coefficient of ABTS^+^ is 36,000 L/(mol·cm). 

In a typical test, 5 mL of ABTS substrate solution at 5 mM concentration in a 100 mM buffer at a given pH was inserted into a 10 mL tube. The reaction was started by addition of 0.5 mL of laccase solution at given pH and concentration. The reaction was usually carried out in an orbital stirrer (100 rpm) at 37 °C. One unit of laccase activity was defined as the amount of laccase required to convert 1 μmol of ABTS substrate per minute under assay conditions.

### 4.5. Degradation of Dyes by Laccase

In a typical test, 5 mL of dye solution at the concentration of 10 mg/L and at given pH, containing 5 mM HBT as a laccase mediator, was placed in a 10 mL tube and inserted in an orbital stirrer at 37 °C. To start the reaction, 0.1 mL of a laccase solution at a given concentration was added. At different time intervals, samples were uptaken and the optical density was measured spectrophotometrically. 

### 4.6. Immobilization of Laccase on Chitosan Hydrogel

The immobilization of laccase on chitosan hydrogel particles was carried out by adsorption. However, as glutaraldehyde crosslinking was carried out for the synthesis of chitosan hydrogels, partially unreacted glutaraldehyde molecules may have contributed to increase the enzyme immobilization. Consequently, the actual mechanism of the laccase immobilization could be described as adsorption/covalent binding. In a typical test, 10 mg of laccase was dissolved in 20 mL of Na-phosphate buffer solution. Subsequently, 200 mg of chitosan particles was added to the solution and kept under gentle stirring (60 rpm) for 24 h at 4 °C. It has been previously ascertained that 24 h is sufficient for the adsorption equilibrium to be reached. At the end of the adsorption processes, the chitosan particles were separated by filtration under vacuum and washed 3 times with Na-acetate buffer, then dried at room temperature. 

The catalytic activity of the immobilized laccase was measured using the method described in Section 4.4.

No appreciable variation in the catalytic activity of the immobilized laccase was observed when incubating the immobilized enzyme in aqueous solution for 48 h at 37 °C adopting different pH values (5, 6, 7, 8, 9).

### 4.7. Adsorption/Degradation of Dyes by Laccase Immobilized on Chitosan Hydrogel

In a typical test, 5 mL of dye solution at given concentration and pH, containing 5 mM HBT as a laccase mediator, was placed in a 10 mL tube and inserted in an orbital stirrer at 37 °C. To start the simultaneous adsorption/catalytic reaction, 50 mg of chitosan hydrogel particles containing immobilized laccase was added to the solution, and kept under stirring (100 rpm). At different time intervals, samples were uptaken. The solid particles were separated by filtration under vacuum, and the optical density of the solution was measured spectrophotometrically.

### 4.8. Reuse of the Immobilized Laccase in Multiple Cycles

The reusability of the laccase immobilized on chitosan was investigated by carrying out the degradation tests at 37 °C. After each cycle, the enzyme was washed with acetate buffer at the same pH used for the degradation test, and separated by centrifugation (5000 rpm, 10 min), to be subsequently reused.

### 4.9. Statistical Analysis

All the experiments were carried out in triplicate. All data are presented as mean ± standard deviation (*n* = 3). 

## Data Availability

Not applicable.

## Supplementary Materials

The following supporting information can be downloaded at: https://www.mdpi.com/article/10.3390/gels9010041/s1; Table S1. fitting parameters for the adsorption of Methylene Blue (MB), Bromophenol Blue (BPB), Coomassie Brilliant Blue (BB) on chitosan hydrogel; Figure S1. Fourier Transformed Infrared Spectroscopy (FTIR) spectra of chitosan (a) and chitosan hydrogel (b).
