# Supplementary material for: A Novel Approach, Based on the Combined Action of Chitosan Hydrogel and Laccases, for the Removal of Dyes from Textile Industry Wastewaters
Source: Gels. 2023 Jan 4;9(1):41. doi: 10.3390/gels9010041 (PMC9857942; doi:10.3390/gels9010041)
Supplement: Supplementary file 1 [file gels-09-00041-s001.zip › gels-2084867-supplementary.pdf]

A novel approach, based on the combined action of chitosan hydrogel and laccases, for the removal of dyes from the textile industry wastewaters

SUPPLEMENTARY INFORMATION

S1. Kinetic models

Three kinetic models were taken into account describe the *adsorption of Methylene Blue (MB), Bromophenol Blue (BPB), Coomassie Brilliant Blue (BB) on chitosan hydrogel:*

- pseudo-first-order (PFO)

$$\frac{dq_t}{dt} = k_{p1}(q_e - q_t),$$

where q_e and q_t are the amount of dye adsorbed (mg g^{-1}) at equilibrium and at time t, respectively, k_{p1} is the rate constant of adsorption (min^{-1}) and t is the time (min). The linear form of the PFO model can be written as:

$$\log(q_e - q_t) = \log q_e - \frac{k_{p1}}{2.303}t.$$

- pseudo-second-order (PSO)

$$\frac{dq_t}{dt} = k_{p2}(q_e - q_t)^2.$$

where k_{p2} is the rate constant of adsorption (g mg^{-1} min^{-1}) and t is the time (min). The linear form of the PFO model can be written as:

$$\frac{t}{q_t} = \frac{1}{V_0} + \frac{1}{q_e}t,$$

where:

$$V_0 = k_{p2}q_e^2,$$

- intraparticle diffusion

The linear form of the intraparticle diffusion model can be written as:

$$q_t = K_{diff} \cdot t^{\frac{1}{2}} + c$$

where K_{diff} is the rate constant of adsorption (mg g^{-1} $min^{-1/2}$) and c is amount of dye adsorbed (mg g^{-1}) at t=0.

Table S1 - fitting parameters for the adsorption of Methylene Blue (MB), Bromophenol Blue (BPB), Coomassie Brilliant Blue (BB) on chitosan hydrogel.

	PFO			PSO			Intraparticle diffusion		
	q_e (mg g^{-1})	K_{p1} (min^{-1})	R^2	q_e (mg g^{-1})	K_{p2} (g mg^{-1} min^{-1})	R^2	K_{diff} (mg g^{-1} min$^{-1/2}$)	c mg g^{-1}	R^2
**MB**	26.4	0.0147	0.918	66.0	8.09 10^{-5}	0.996	2.68	24.4	0.861
**BPB**	32.1	0.0139	0.947	64.9	1.36 10^{-4}	0.990	3.03	15.1	0.876
**BB**	22.1	0.0159	0.898	45.7	3.79 10^{-4}	0.989	2.16	10.6	0.871

S2. Characterization of the chitosan hydrogel by Fourier Transformed Infrared Spectroscopy (FTIR)

The infrared spectra of the chitosan hydrogel were obtained using a FTIR spectrometer (Thermo Nicolet, USA).

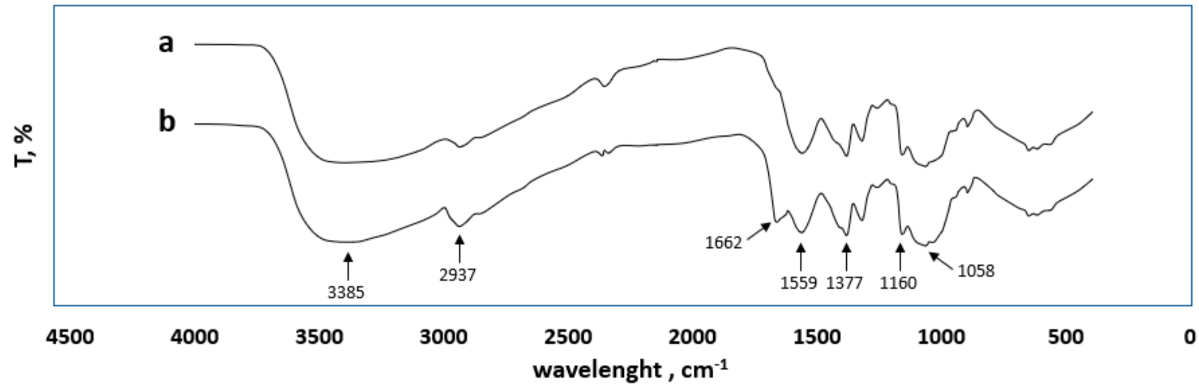

Figure S1. Fourier Transformed Infrared Spectroscopy (FTIR) spectra of chitosan (a) and chitosan hydrogel (b)

The FTIR spectra of the chitosan hydrogel, shown in the Fig. S1 (curve b), indicated the presence of the of the functional groups described in the following. The peaks (Typical bands) at 3385 cm^{-1} and 2937 cm^{-1} correspond to the stretching of O-H and of C-H, respectively. The peak at 1559 cm^{-1} can be attributed to the N–H group. The –CH_3 symmetrical deformation was confirmed by the peak at 1377 cm^{-1}. The peak at 1160 cm^{-1} can be attributed to the asymmetric stretching of the C-O-C bridge. The peak at 1058 cm^{-1} corresponds to the stretching of C–O group.

All these peaks can be observed to some extent also in the chitosan spectrum (curve a), that has been reported as reference. Yet, the effective formation of the chitosan hydrogel is demonstrated by the presence of the peak at 1662 cm^{-1} (not found in the curve a), corresponding to the imine groups (C=N) formed by covalent bonding between the free amino groups of chitosan and the aldehyde groups of glutaraldehyde.
